# Cystitis

**Published:** 1905-07

**Authors:** E. G. Ballenger

**Affiliations:** Atlanta, Ga.


ATLANTA

### Journal-Record of Medicine.

Successor to Atlanta Medical and Surgical Journal, Established 1855, and Southern Medical Record, Established 1870.

OWNED BY THE ATLANTA MEDICAL JOURNAL CO.

Published Monthly.

Vol. VII. JULY, 1905. No. 4.

BERNARD WOLFF, M.D., EDITOR, Nos. 319-20 Prudential.

M. B. HUTCHINS, M.D., BUSINESS MANAGER, 1007-1008 Century Bldg.

J. N. LeCONTE, M.D., ASSOCIATE EDITOR.

### ORIGINAL COMMUNICATIONS.

### CYSTITIS.*

By E. G. BALLENGER, M.D.,
Atlanta, Ga.

The rather complex etiology and pathology of this condition, on account of its close relation to nearly all the important genito-urinary diseases, prevents more than a brief discussion of the essential features.

The brilliant advances in renal and prostatic surgery in recent years, in addition to the great improvements in the diagnosis of all bladder diseases that have been made possible by the cystoscope and microscope, have cleared up many of the doubtful points in cystitis, nevertheless there are several important questions that can only be determined by careful routine examination in the future. These contentions will not be discussed, but the consensus of opinion given.

*The Etiology* is apparently dependent upon two conditions:

1st. A predisposition.

2d. A pyogenic infection.

*Read before the Atlanta Society of Medicine June 1, 1905.

The exact manner in which all the predisposing causes act is not entirely understood. The most frequent and certain of the conditions that tend to cause cystitis are stricture, prostatic hypertrophy, cystic calculi and neoplasms, congestion of pelvic viscera from any cause whatever, traumatism, foreign bodies, instruments, parasites, uterus displaced anteriorly, exposure to cold, strong diuretics, balsams, cantharides, turpentine, urethritis, gout, diabetes, nervous diseases and especially those involving the spinal cord, pareses of the insane and workers in hydrochloric acid and other acids (1). All of these are very important because the persistence and intensity of a cystitis is proportionate to the predisposition.

The promptness of a cure under appropriate treatment therefore depends upon removing the predisposing cause, for there is always a tendency for the suppurative condition to clear up if this is removed. Retained urine and the congestion of the mucous membrane that attends it furnish a most favorable soil for the growth of micro-organisms. Most authorities agree that ammoniacal decomposition is an effect and not a cause of cystitis.

Lowered resisting power from systemic weakness makes it possible for microbes to flourish and produce a severe inflammation. There is, however, no such thing as "idiopathic catarrh of the bladder."

Under fovorable conditions any pyogenic organism may set up a cystitis. Those most frequently encountered are the bacillus coli communis and this group, streptococcus, staphylococcus aureus, albus and citreus, several varieties of the proteus, bacillus pyocyaneus, bacillus lactis aërogenes, micrococcus ureae and a rare form which liquefies urea.

The position of the gonococcus has not been definitely settled. The general opinion is that there are a certain number of cases where it is the actual cause, but in the majority it seems to pave the way for some other pyrogenic microbe. The infection with the tubercle bacillus very frequently starts from a chronic gonorrhoea, especially where it has been neglected.

The staphylococcus citreus and the proteus vulgaris, in addition to the micrococcus ureae and this type of saprophytic micro-organisms have the power of decomposing urea.

There are a number of infectious diseases as scarlet fever, typhoid fever, diphtheria, etc., in which the germs are apparently eliminated through diseased points in the kidneys and inflammation of the bladder occurs during the height of the disease or during convalescence. The urine may be swarming with bacteria without any evidence of a cystitis and patients have gone for years secreting urine full of tubercle bacilli from the kidneys without causing a cystitis. This, however, is the most frequent route for tubercular infection while nearly all suppurative cystites are caused by infection entering through the healthy or diseased urethra.

Kelly (2) claims that, in women who have borne many children and have patulous urethras, infection may travel up from the vulva to the bladder, while Swart (3) believes that cystitis in children, especially in girls, is caused by the colon bacillus which migrates from the rectum into the urethra. He urges that in children suffering from enteritis especial care be taken to avoid soiling the vulvar parts with the fecal discharges. Guyon (4) has shown that the microbes are attenuated by frequent emptying of the bladder and its occasional disinfection and advises that all operations be postponed, when possible, until the germs are in this state of hypovirulence.

Westphal (5) reports a case of cystitis caused by the malarial plasmodia.

*Pathology.* The simple catarrhal cystitis shows an hyperemia of the mucous membrane which may be coated with tenacious mucous, rich in pus-cells and epithelium (6). This usually begins in the neck and trigone where the inflammation is most severe. The epithelium is cloudy and swollen.

In chronic inflammation the formative changes are very marked and the mucosa is thickened by a round cell infiltrate which gradually changes to fully formed connective tissue.

Hemorrhage in chronic cystitis as a rule means there is an ulceration.

Membranous cystitis is not so rare and is seen in cord disease or injury from pressure during childbirth and from excessive distention of the bladder. Ulcers are comparatively frequent in both acute and chronic conditions and are usually located in the base or around the urethral orifices.

Phlegmonous and gangrenous cystites are very severe in character. Their names indicate the conditions that exist.

Hypertrophy of the muscular coat occurs in all chronic cases. This is not regular but ridges stand out, giving the bladder a corrugated appearance. If this condition of thickening progresses and lessens the bladder cavity it is called concentric hypertrophy. Fatty degeneration and retention of the urine may result in distortion which is called excentric hypertrophy. The mucous membrane frequently pouches through the weak points forming sacculations. These are favorite seats for stones.

Tubercular lesions begin as modules in the mucous membrane which caseate and finally form ulcerations. It is not until this stage that tubercle bacilli are found in any numbers.

*The Symptoms* may be constitutional in acute cases and in chronic cases where toxins are absorbed from the diseased bladder. Pain is nearly always found but varies much in character. It is usually worse when the bladder is full or completely empty or during the passage of urine if the inflammation is severe or there is any obstruction. The pain of tubercular cystitis is very similar to stone and many times secondary infection is started by a careless exploration. There is tenderness on palpating above the pubes or through the rectum in acute cases, but it is less marked in chronic and tubercular patients. Frequency of urination is always found and varies from a slight increase to a constant desire, only passing a few drops after great straining. Frequency may depend upon one or more of several causes, as very severe inflammation, especially of the vesical neck, retention of part of urine each time or lessening of the cavity of the bladder by concentric hypertrophy. Pus in the last glass is due to cystitis if the kidneys are not involved.

*Diagnosis.*—When taken with pain, frequency and tenesmus, the diagnosis is certain, but it may not be limited to the bladder, hence the importance of locating its primary origin. A stone impacted low down in the ureter gives symptoms very similar to cystitis (7).

Inflammation of the bladder in addition to pus furnishes mucuos, bladder epithelium, red blood-cells and usually a heavy crystalline deposit in the urine. It has a cloudy appearance, and if allowed to stand, a whitish precipitate settles to the bottom, leaving the urine above clear if acid, but this remains cloudy if alkaline, and the cells and casts are altered in appearance. The free ammonia decomposes the pus, forming a gelatinous substance very similar to and often mistaken for mucus. This may block the passage and cause collicky pains. The urine has a foul, disagreeable odor in the severe forms. Albumen is always found and Posner claimed that 100,000 pus cells per c. m. could only account for 1 per cent of albumin and estimated from this whether or not the kidneys were involved. Casts are also important evidence of renal disease. In all cases a microscopic examination of the urine should be made, and in a certain number where the symptoms are not clear or do not yield to ordinary treatment a cystoscopic examination is advisable to locate accurately the primary trouble and to direct rational treatment toward its removal.

To Nitze belongs the credit of discovering and perfecting the cystoscope. The modifications are too numerous to mention. It is one of the most important adjuncts in the diagnosis and treatment of diseases of the bladder.

For a successful examination the urethra and prostate must admit the easy introduction and manipulation of the cystoscope. The bladder must hold from 90 to 120 grams of fluid and bleeding must not be so profuse as to cloud the view. It is counter-indicated in acute conditions of the bladder, uretha or kidneys and in inoperable conditions that may be made worse by it. Tuberculous cystitis has increased in importance as examinations have been more carefully made. It is most important to make a distinction between chronic cystitis caused by pus microbes and that caused by tubercle bacilli. Many serious mistakes are made in the prognosis and treatment of cases not properly diagnosed. The existence of a chronic inflammation of the bladder in the absence of tangible evidence of infection with gonorrhœa, chronic urinary obstruction or infection from instrumentation should always leave a suspicion of a tubercular condition (8).

The urethra, prostate, seminal vesicles in men and the uterine appendages in women should always be carefully examined. The urine should be studied repeatedly by centrifugation and staining as well as by inocculation experiments to find tubercle bacilli. The tuberculin test is fairly reliable, the symptoms, pain frequency and tenesmus being made temporarily worse.

The differential diagnosis is especially important if cystotomy is contemplated, for the good results that follow in an intractible cystitis due to pyogenic infection can not be expected in tubercular conditions.

Hematuria may be very early and intermittent in tubercular cystitis, but it is never profuse as in tumors of the bladder. Usually there are a few drops of blood at the end of the act.

*The Prognosis* depends upon the predisposing cause and the kind of infection. The colon bacillus seems more likely to invade the urethra and kidneys. Tubercle bacilli are of course the most intractable. The cure depends upon removing the predisposing cause, as stricture, enlarged prostate, stone, neoplasm, etc., and upon the extent of alterations in the bladder walls. In the majority of cases, if the primary cause is found and removed and the proper medication, diet and hygiene carried out, a cure may be expected except in the gangrenous and phlegmonous cystites. The tubercular can be modified, the patient made more comfortable and life prolonged, but a complete cure can rarely be obtained. The importance of a correct diagnosis is evident before making any promises as to the probable results.

*Treatment.*—In acute cystitis the chief indications are, rest in bed, light diet, bowels kept open, urine rendered bland and unirritating by copious draughts of water, milk, buttermilk, whey and lithia water. Hot fomentations to the pelvis or hot sitz baths lessen the congestion and relieve the pain. In women hot vaginal douches should be given once or twice daily. These, as a rule, are sufficient to make the patient comfortable, if not, suppositories may be given.

℞ Morphine sulphate.................................grs. 1-12.
Ext. belladonn.
Ext. hyocyam...............................aa. gr. 1-4.
Cocoa butter.......................................grs. xii.
M. et ft. suppos. No. 1.

Sig. One such introduced into the rectum every few hours as required for the pain.

Urotropin or cystogen in 5 to 10-grain doses are valuable to make the urine antiseptic, but are particularly indicated when the urine is alkaline.

Irrigations should not be given in hyperacute cystitis or if there is an acute urethritis or nephritis, but may be used with decided benefit in the subsiding stage to prevent the cystitis from becoming chronic. The fluid should have about the same specific gravity as the urine. The following has given very satisfactory results :

℞ Boric acid.

Borax........................................aa. ʒi.

Sodium chloride..............................gr. xxx.

M.

Sig. Dissolve in a quart of hot water and use as irrigation. The best method of washing out the bladder is with a glass funnel attached to 6 feet of rubber tubing and connected with a glass catheter in women, or a soft rubber one in men. The catheter after thorough sterilization is introduced and by lowering the funnel the urine flows out and fills it. The tube is compressed and the urine replaced by the irrigating fluid which flows into the bladder when the funnel is raised. This prevents the introduction of air which causes pain. The bladder is washed until no shreds, pus or mucous can be seen.

In men the bladder may be irrigated in a very satisfactory manner with the apparatus used for urethral irrigation, using a blunt pointed nozzle. These should be repeated once or twice daily or discontinued if they are not well borne.

As the disease becomes chronic the irrigations should be made more stimulating, as mercuric chloride 1-5000 to 1-800, potassium permanganate 1-12000 to 1-1000, or nitrate of silver 1-4000 to 1-800.

Begin with a weak solution and gradually increase as the bladder will tolerate. The chief good, however, comes from the cleansing rather than the stimulating action.

In the cases of urethro-cystitis, where the pain and tenesmus are very severe, prompt relief may be obtained by instillation of 15

to 20 M of 4 per cent. cocaine or 1 to 4 per cent. nitrate of silver. This should be injected beyond the compress or urethræ muscle.

The treatment of tuberculous cystitis is largely systemic to increase the vitality of the tissues.

Guiacol and creosote gtts. v-xv of each and continued for a long time may do some good. Tonics, iron, and laxatives should be administered as indicated; cod-liver oil, change of climate, rich, nourishing food, plenty of fresh air, sunshine and regulated exercise may all be of service. Bicycle and horseback riding are always harmful. Local injections require great pains to prevent secondary infection. Five to ten per cent. iodoform in an emulsion of olive oil and glycerine left in the bladder may be beneficial. The patient is instructed to watch the flow of urine and stop when the emulsion appears.

Tumors should be removed when diagnosed. If the cavity of the bladder is small, its capacity may be increased by over-filling with irrigations or by resolutely holding the urine until the bladder is somewhat dilated. These methods should never be employed in the tubercular cystitis. Balsam capaiba and oil of sandal-wood have a stimulating action and may be given in from 5 to 10-grain doses in chronic catarrhal cystitis. Hot air boxes, sulphur and salt baths may also be of value.

Chronic ulcers should be touched, through an endoscope, with nitrate of silver fused on a probe. Frequently a few treatments will be sufficient to cure.

If a calculus is present it can be crushed and removed through the urethra or by cystotomy as conditions require.

Hypertrophy of the prostate, if small and fibrous without much vesical enlargement, should be removed by the perineal operation. With the large adenomatous ones, that are more easily shelled out of the sheath, the supra-pubic route is preferred.

Strictures are treated by gradual dilation with plain sounds followed by those covered with a coating of cocoa butter containing nitrate of silver, or the medication desired, to allay the chronic urethritis that is always found in these cases. The ointment is massaged into the follicles and depressions of the mucous membrane. This treatment combines more factors working towards a cure than any other single method, and as I have mentioned it several times before in the society, I have prepared some sounds to show how they are used. For eighteen months they have proven most satisfactory in the treatment of stricture, chronic urethritis and chronic prostatitis. Massage of the stricture hastens the absorption of the abnormal tissues.

From some recent studies and examinations I am confident when the method of dilation fails that the most rational treatment with the most permanent result will be an external operation, making a longitudinal incision into the urethra at the stricture and sewing it up transversely to increase the caliber at this point. This method is certainly less formidable than total excision of the strictures as performed by many surgeons of repute.

BIBLIOGRAPHY.

1. Spooner, *Medical Record*, New York, December 5th, 1903.
2. Kelly, Operative Gynecology, 1899.
3. Swart. Medizinskoe Oborsenie, LXIII, No. 2.
4. Guyon. Presse Med. Paris, Vol. 93, No. 19, 1904.
5. Westphal, New Orleans *Medical and Surgical Journal*, July 1904.
6. Lydston, American T. B. Genito-Urinary Surgery.
7. Fowler, Annals of Surgery, December, 1904.
8. Senn, Genito-Urinary Tuberculosis, 1897.

---

### A REMEDY FOR FACIAL ERYSIPELAS.

Dr. E. S. Breese says: "As soon as a diagnosis of erysipelas is made a calomel purge is given, tincture of chlorid of iron in moderate doses ordered every three hours, and ichthyol and tincture of iodin, equal parts, painted well over the diseased area. The local application is made daily, care being taken to go beyond any spreading border. The symptoms usually abate in from three to five days. The epidermis of the painted area exfoliates. The temperature seldom becomes high, and delirium occurred only twice. This remedy is mentioned because it has been tried and acts almost like a specific."—*Dietetic and Hygienic Gazette.*

